# Predictive Value of Diminished Serum PDGF-BB after Curative Resection of Hepatocellular Cancer

**DOI:** 10.1155/2019/1925315

**Published:** 2019-01-06

**Authors:** Bibek Aryal, Munekazu Yamakuchi, Toshiaki Shimizu, Jun Kadono, Akira Furoi, Kentaro Gejima, Teruo Komokata, Chihaya Koriyama, Teruto Hashiguchi, Yutaka Imoto

**Affiliations:** ^1^Cardiovascular and Gastroenterological Surgery, Graduate School of Medical and Dental Sciences, Kagoshima University, Kagoshima 890-8520, Japan; ^2^Department of Laboratory and Vascular Medicine, Graduate School of Medical and Dental Sciences, Kagoshima University, Kagoshima 890-8520, Japan; ^3^Department of Surgery, Kirishima Medical Center, Kirishima 899-5112, Japan; ^4^Department of Surgery, Kagoshima Medical Center, National Hospital Organization, Kagoshima 892-0853, Japan; ^5^Department of Epidemiology and Preventive Medicine, Graduate School of Medical and Dental Sciences, Kagoshima University, Kagoshima 890-8520, Japan

## Abstract

**Purpose:**

Platelet derived growth factor-BB (PDGF-BB) has emerged as one of the key cytokines in malignant transformation of different cells. PDGF-BB also exhibits a potent mitogenic effect on liver cells; studies have advocated clinical implications of monitoring serum PDGF-BB (sPDGF-BB) in patients with liver disease. We thus investigated the predictive relevance of perioperative sPDGF-BB after curative resection of hepatocellular carcinoma (HCC).

**Methods:**

We evaluated perioperative sPDGF-BB in a prospective homogenous cohort of 40 patients diagnosed with HCC. During the first two-year follow-up, patients were evaluated every three months for postresection HCC recurrence.

**Results:**

Patients who developed recurrence during two-year follow-up were found to have lower concentration of sPDGF-BB than those without recurrence in both pre- and postoperative settings (*P* < 0.05 and* P* < 0.001, resp.). We validated that the reduced postoperative sPDGF-BB (< 2133.29 pg/mL) was associated with an increased incidence of postresection HCC recurrence [area under curve (AUC) > 0.8, 95% confidence interval (CI) = 0.68 - 0.94,* P* < 0.001]; furthermore, we were able to demonstrate that postoperative sPDGF-BB was an independent predictor of HCC recurrence (hazard ratio = 5.64, 95% CI = 1.56 - 20.30,* P* < 0.01).

**Conclusions:**

These findings provide a new insight into an association between diminished perioperative sPDGF-BB and HCC recurrence. Patients with low perioperative sPDGF-BB progressed early HCC recurrence. Therefore, evaluating perioperative sPDGF-BB may provide useful clinical information to characterize patients with postresection HCC recurrence.

## 1. Introduction

Curative resection (partial hepatectomy) is a safe, effective, and preferred therapy for selected patients with hepatocellular carcinoma (HCC)[1]. However, the tumor recurrence particularly in the remnant liver is exceedingly frequent after the surgical resection of HCC[2]. The high incidence of HCC recurrence intensifies the need for identifying high-risk patients, ideally before the recurrence sets in, by integrating both clinical and molecular information into a synergistic understanding of the tumor recurrence.

Platelet-related biomarkers, detected in the circulation (whole blood, serum, or plasma), have long been considered as a potential diagnostic tool in cancer[3–7]. These growth factors have demonstrated several implications in the field of oncology including screening, diagnostic, and prognostic relevance of diseases[7]. Platelet-related growth factors reflect paradoxical association with different physiological and pathological events[8]. For instance, the platelet-sequestered vascular endothelial growth factor (VEGF), which is linked to various pathological conditions[9] including carcinogenesis, has also been found to be crucial in physiological events like liver regeneration after partial hepatectomy[10, 11].

Growing lines of evidence recognize the role of platelet derived growth factor-BB (PDGF-BB) at every stage during the continuum of liver injury, repair, and fibrosis[12–14]. Despite a wealth of literature on PDGF-BB in other tumor types, there are relatively a few studies on the PDGF-BB and its predictive relevance in HCC. Also, to date, no evidence examining the feature of serum PDGF-BB (sPDGF-BB) on postresection HCC recurrence has been published in English literature. In this study, we aimed to determine whether the perioperative sPDGF-BB could reflect the oncological outcome after the curative resection of HCC.

## 2. Materials and Methods

### 2.1. Study Cohort

The study cohort consisted of forty patients diagnosed with primary HCC who went on to have liver resection. The trial is registered in UMIN Clinical Trial Registry (UMIN000026380). The institutional ethics committee (Kagoshima University # 24-155/ 26-77, Kirishima Medical Center # 2505 and Kagoshima Medical Center # 25-30) approved analyses of blood samples and patient data; all patients gave signed, informed consent.

### 2.2. Follow-Up

Disease-free interval (DFI) was defined according to the Guidance for Industry Clinical Trial Endpoints for the Approval of Cancer Drugs and Biologics of the US Food and Drug Administration (FDA). DFI represents the time period between liver resection and tumor recurrence. Follow-up period was standardized to two years; patients were followed up every three months after surgery. Follow-up was routinely scheduled and comprised of ultrasonography (USG) as well as evaluation of tumor markers; if USG showed any evidence of tumor, further assessment of thoracic and abdominal CAT scans or MRI was performed. Tumor recurrence was diagnosed based on analysis of radiological findings and comprised of local and distant recurrence.

### 2.3. Sample Preparation

Venous blood was collected preoperatively, immediately before surgery (PRE OP), and four weeks after surgery (POST OP). Complete blood count (CBC) was performed with an automated hematology analyzer Sysmex XE-5000 (Sysmex Corporation, Kobe, Japan).

Whole blood was collected in the serum-separating tube and an EDTA-2k. Serum tube was incubated at room temperature for 30 minutes before centrifuging at 1710 × g for 10 minutes.

### 2.4. Platelet Extract

Venous blood in citrate tubes was centrifuged at 90 × g for 15 minutes. To avoid contaminations with other cells, upper two-thirds of the resultant platelet-rich plasma (PRP) were gently pipetted. The PRP was then centrifuged at 2810 × g to isolate platelets. The supernatant, platelet-poor plasma (PPP), was collected precisely and removed completely by decantation method. Platelet pellets isolated from each 200 *μ*l of PRP were suspended in 220 *μ*l of lysis buffer (RIPA) and were vortexed after incubating for 20 minutes. CBC was performed in 3 preparations: whole blood, PRP, and PPP.

All samples were stored in aliquots at -80°C immediately after preparation.

### 2.5. Quantification of Cytokines

Platelet derived growth factor-BB and P-selectin were measured using enzyme-linked immunosorbent assay (ELISA) tests (Quantikine; R&D Systems, Minneapolis, MN, USA). Intraplatelet (IP) PDGF-BB was calculated from isolated platelets using the equation to calculate cytokines inside each platelet, as described before[10]. Concisely, 220 *μ*l of lysis solution (RIPA) was added to the platelets isolated from each 200 *μ*l of PRP. The concentration of the cytokine was adjusted to the platelet count obtained from the PRP and IP PDGF-BB was expressed per 10^6^ platelets.

### 2.6. Statistics

Statistical analyses were performed using SPSS 25.0 software (SPSS, Inc., Chicago, IL, USA) and GraphPad Prism (version 6.0d for MacOS X, USA, GraphPad Software, San Diego, California, USA) and were mainly based on nonparametric tests (Mann-Whitney* U* test and Wilcoxon test). Receiver operating characteristic (ROC) analysis was performed to assess the specificity and sensitivity of PDGF-BB levels to predict recurrence. Youden's J index was applied to determine cut-off points in ROC analyses. Cox's proportional hazards regression model was used for the univariable analyses (UVA) and multivariable analyses (MVA) to determine the variables independently associated with recurrence. Two-tailed* P* values of less than 0.05 were considered statistically significant. IP PDGF-BB and IP P-selectin concentrations were expressed per 10^6^ platelets.

## 3. Results

### 3.1. Patient Demographics

We included forty patients with HCC who were selected for liver resection. Baseline characteristics of the study cohort are summarized in Table 1. None of the patients received platelet transfusion during the sample preparations. During two-year follow- up, 15 patients developed HCC recurrence and one patient died with a cause other than the cancer recurrence.

### 3.2. Perioperative sPDGF-BB Concentrations Were Lower in Patients with HCC Recurrence

**Figure 1:**
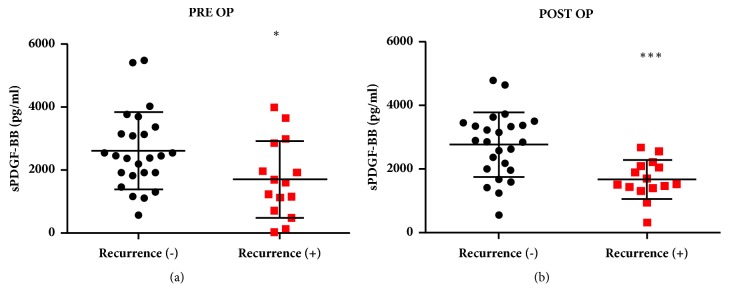
Serum PDGF-BB (sPDGF-BB) concentration in recurrent and nonrecurrent cases (a) before (PRE OP) and (b) 4 weeks after liver resection (POST OP). ^*∗*^P< 0.05 and ^*∗∗∗*^P< 0.001.

We first compared perioperative sPDGF-BB concentrations in patients with postresection HCC recurrence. Patients with two-year postresection HCC recurrence had significantly lower concentrations of sPDGF-BB prior to liver resection (PRE OP median sPDGF: nonrecurrent, 2444 pg/mL; recurrent, 1592 pg/mL,* P* = 0.02; Figure 1(a)) and after four weeks of liver resection compared to the patients with no cancer recurrence (POST OP median sPDGF: nonrecurrent, 2852 pg/mL; recurrent, 1592 pg/mL,* P* < 0.001; Figure 1(b)).

Furthermore, we compared separately isolated, intraplatelet (IP) concentrations of PDGF-BB in patients with and without recurrence. We observed similar but a weak trend; patients with HCC recurrence had lower IP PDGF-BB concentrations preoperatively (PRE OP median IP PDGF-BB: nonrecurrent, 12.38 pg/10^6^ platelets; recurrent, 8.94 pg/10^6^ platelets,* P* = 0.709; Fig S1A) and after four weeks of liver resection (POST OP median IP PDGF-BB: nonrecurrent, 12.43 pg/10^6^ platelets; recurrent, 9.24 pg/10^6^ platelets,* P* = 0.43; Fig S1B). The difference in IP PDGF-BB between recurrent and nonrecurrent cases did not yield a statistically significant result.

We also examined the possibility of increased platelet activation in patients with or without recurrence by evaluating the total platelet P-selectin in patients with or without recurrence and found no substantial difference in the concentrations of total platelet P-selectin between the groups (PRE OP median IP P-selectin: nonrecurrent, 520.7 pg/10^6^ platelets; recurrent, 507 pg/10^6^ platelets,* P* = 0.709; Fig S2A, and POST OP Median IP P-selectin: nonrecurrent, 477 pg/10^6^ platelets; recurrent, 455 pg/10^6^ platelets,* P* = 0.61; Fig S2B).

### 3.3. Perioperative sPDGF-BB Values Specifically Characterize Patients with Postresection HCC Recurrence

**Figure 2:**
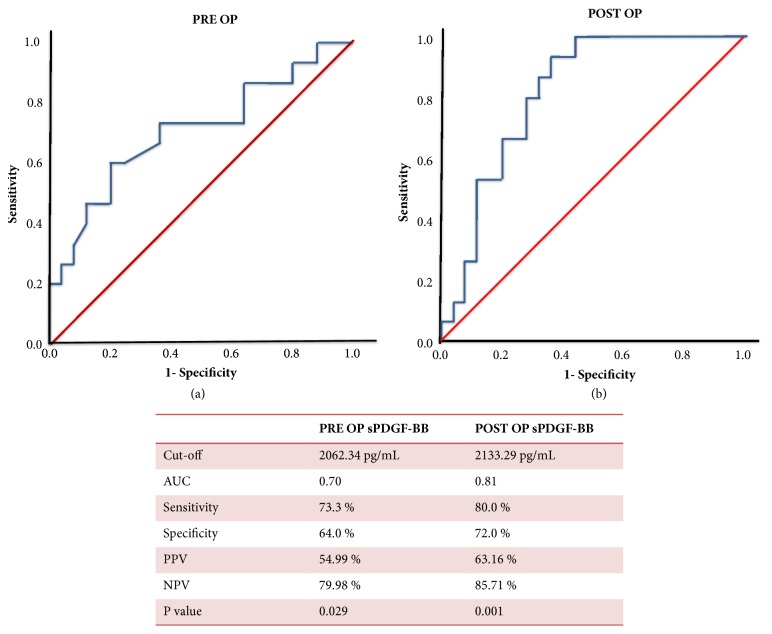
Receiver operating characteristic curve for the preoperative (a) and postoperative (b) serum PDGF-BB (sPDGF-BB).

Given the substantial difference in concentrations of sPDGF-BB in patients with and without postresection HCC recurrence, we further focused on its clinical relevance. First, we determined a cut-off point for both pre- and postoperative sPDGF-BB by using the receiver operating characteristics (ROC) curves (Figures 2(a) and 2(b)). We then applied Youden's J index and identified a cut-off value of 2062.34 pg/ml for PRE OP sPDGF-BB [area under curve (AUC): 0.709, sensitivity: 73.3%, specificity: 64.0%, 95% confidence interval (CI) = 0.53 – 0.88,* P* = 0.029] and 2133.29 pg/ml for POST OP sPDGF-BB (AUC: 0.816, sensitivity: 80.0%, specificity: 72.0%, 95% CI = 0.68 – 0.94,* P* = 0.001) to distinguish between the high- and low-risk patients in terms of HCC recurrence. This translates to the positive predictive value (PPV) of 54.99% and negative predictive value (NPV) of 79.98% for preoperative sPDGF-BB and similarly PPV of 63.16% and NPV of 85.71% for postoperative sPDGF-BB.

### 3.4. Postoperative sPDGF-BB Independently Predicts Postresection HCC Recurrence

We observed distinctly depleted perioperative concentrations of sPDGF-BB levels in patients with postresection HCC recurrence; furthermore, it was of interest to investigate if sPDGF-BB could independently predict postresection recurrence. Therefore, we first performed univariable (UVA) and multivariable survival analysis (MVA) using Cox's proportional hazard model. Among the 4 significant variables in UVA, we excluded the postoperative platelet count in MVA because of its collinearity to all other 3 variables.

As seen in Table 2, postoperative sPDGF-BB was able to independently predict the postresection recurrence (hazard ratio = 5.64, CI = 1.56 - 20.30,* P* = 0.008).

**Figure 3:**
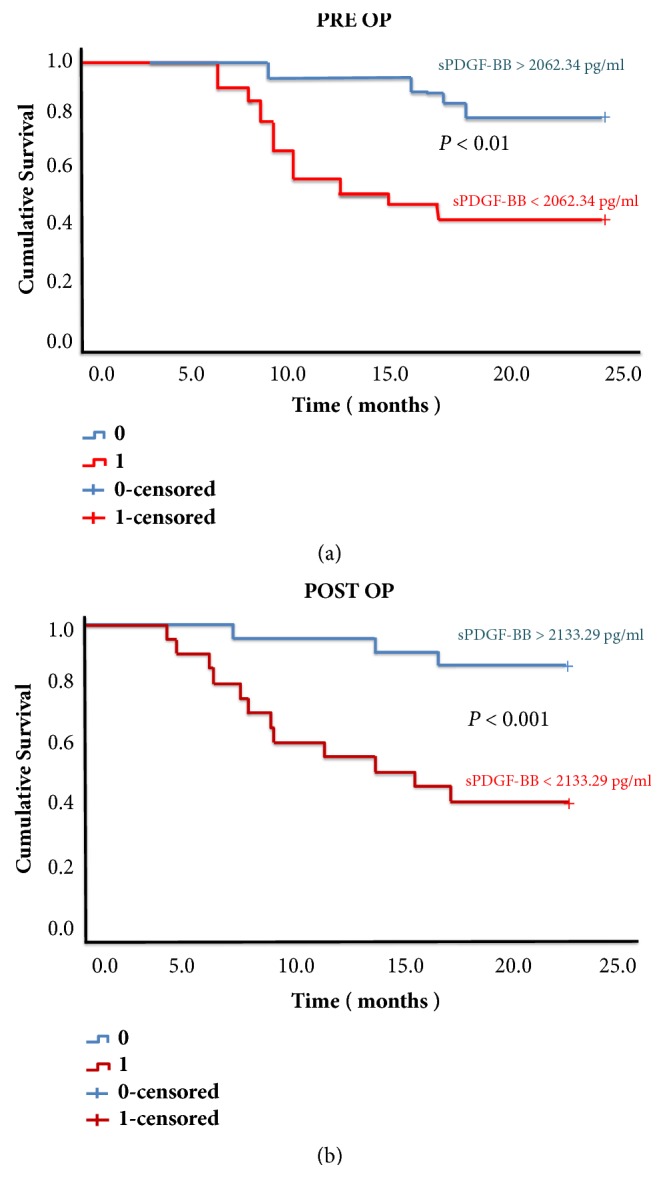
Kaplan-Meier disease-free survival curves based on preoperative (PRE OP) (a) and postoperative (POST OP) (b) serum PDGF-BB (sPDGF-BB) concentration.

Furthermore, a log rank test was run to determine the differences in disease-free interval (DFI) distribution between groups with high and low perioperative serum sPDGF-BB. The Kaplan-Meier curves generated distinct DFI pattern between the patients with high and low perioperative PDGF-BB; patients with lower preoperative [*χ*2 (2) = 5.97,* P* = 0.01] or postoperative [*χ*2 (2) = 11.04,* P* = 0.001] sPDGF-BB demonstrate shorter DFI (Figures 3(a) and 3(b)).

## 4. Discussion

The overexpression or mutations of PDGF and its prognostic relevance observed in different cancers[15, 16] prompted us to investigate if soluble (serum) PDGF-BB could provide useful information to diagnose HCC recurrence. We detected a substantially lower concentration of perioperative PDGF-BB in the serum of patients with early postresection HCC recurrence.

In this study, we evaluated perioperative PDGF-BB concentration in serum, as serum concentrations also represent PDGF-BB pool stored in platelets. Platelet-stored PDGF-BB is easily available for the tumor and surrounding stroma[14]; thus sPDGF-BB reflects the magnitude of proangiogenic influence related to the growth factor. Platelet dysfunction is already a known phenomenon in cancer progression; platelets hyperactivity and platelet exhaustion are frequently seen in cancer patients[17–20]. We previously observed exhaustion of IP serotonin in patients with early cancer recurrence[17]; likewise, we speculated that the depleted sPDGF-BB might attribute to the platelet exhaustion; however, we observed a weak trend with total platelet (IP) PDGF-BB. Furthermore, no remarkable difference in total platelet P-selectin, observed in the study, rules out the possibility of a varied pattern of platelet activation in patients with or without recurrence. It is clearly known that PDGF-BB in serum is not a soul reflection of PDGF-BB content in platelets; a variety of cells secrete PDGF-BB [13–15]. Thus, the exhaustive behavior of PDGF-BB in serum is not fully attributed to the platelet kinetics in HCC recurrence.

We believe that this is the first report on depleted serum concentrations of PDGF-BB in patients with cancer (HCC) recurrence. Thus, the query rises about the molecular mechanism involved in the exhaustion of sPDGF-BB concentrations in patients with recurrence. Based on our finding, we can speculate that decreased synthesis or increased degradation of PDGF-BB (or both) may occur during the process of HCC recurrence. Some other functional studies have stressed on the response of tumor vessels pericyte to PDGF-BB[21–23]. Frequently irregular and disorganized vascular networks in tumor largely influence the tumor progression and outcome. Intriguingly, endothelial, and not tumor, cells production of PDGF-BB is essential for proper pericytes installation and coverage and formation of stable pericyte-endothelial cell contacts[21]. In a previous study, ablation of pericytes by anti-PDGF agents augmented vascular tortuosity and tumor growth, suggesting a negative role of anti-PDGF-BB-induced pericyte loss in tumor angiogenesis and growth[22]. The same study revealed a dose-dependent effect of PDGF-BB in tumor vessels pericyte loss and demonstrated a detrimental effect of substantially lower PDGF-BB in tumor progression [22]. Stabilization of tumor blood vessel by pericyte is suggested to be a desirable therapeutic goal where new vessel may inhibit formation and tumor growth thereby arrested[21]. Similarly, PDGF-BB-induced pericyte detachment was observed in two different cancer cell lines[22]. In another study by McCarty et al., overexpression of PDGF-BB decreased colorectal and pancreatic cancer growth by increasing the tumor pericyte content [23]. The authors observed that the tumor with highest PDGF-BB expression in vitro had the slowest tumor growth rate in vivo. Another compelling evidence on opposing effects of PDGF-BB levels in tumor microenvironment warrants a caution while selecting the patients for anti-PDGF drugs[22]. These mechanisms somehow justify the findings of our study; a similar phenomenon such as the increased proportion of cancer cells leaking through the less stabilized tumor vasculature in patients with low PDGF-BB could have led to early postresection HCC recurrence.

Investigations on liver fibrogenesis, particularly with the identification of the role played by PDGF on hepatic stellate cells (HSC), have clearly suggested PDGF as the most potent mitogen in the series of tissue repair process after liver injury[24, 25]. The dimeric form of PDGF including at least one B chain (PDGF-AB and PDGF-BB) was found to be more potent for HSC with predominant expression of PDGF-R *β* subunits[26]. The “good” prohealing action of PDGF-BB observed earlier has now been translated for clinical applications, establishing an inverse association between sPDGF-BB concentrations and advanced fibrosis[14]. Takayema et al. observed an association between decreased concentration of sPDGF-BB and poor outcomes in patients with fulminant hepatic failure (FHF) suggesting a protective role of PDGF-BB in liver repair[27]. This observation, to some extent, rationalizes the peculiar results we obtained in our cohort; however, we did not observe a significant association between fibrosis stage and sPDGF-BB concentrations. Nevertheless, the ubiquitous mechanism of fibroproliferative response interlaced with PDGF-BB and the resultant phenotypic change that may occur during the process of cancer recurrence cannot be completely ignored.

Our results provide first in-human evidence for an inverse association between perioperative sPDGF-BB and postresection HCC recurrence. Given the relatively small sample size of this pilot study and with no direct mechanistic evidence, it is difficult to discern what role, if any, sPDGF-BB exhaustion may directly play in the course of postresection HCC recurrence. Yet, this study apprises new clinical evidence on the opposing association of soluble PDGF-BB in cancer recurrence. Further studies with rigorous evaluation including analytical and clinical validation and assessment of clinical utility are essential before incorporating the proposed predictive relevance of diminished sPDGF-BB into the clinical setting.

## Figures and Tables

**Table 1:** Demographics and clinical and pathological data of the cohort.

**Variables**	**Data**
**Age**, median (min, max)	72.52 (51, 83)

**Sex,** n (%)	

Male,	30 (75 %)
Female	10 (25 %)

**Child Pugh Class,** n (%)	

A	40 (100 %)
B	0
C	0

**Etiology,** n (%)	

HBV	12 (30 %)
HCV	13 (32.5 %)
Nonviral	15 (37.5 %)

**Fibrosis grade,** n (%)	

0-2	22 (55 %)
3-4	18 (45 %)

**Tumor staging,** n (%)	

I-II	28 (70 %)
III-IV	12 (30 %)

**Tumor size,** n (%)	

< 5 cm	27 (67.5 %)
≥ 5 cm	13 (32.5 %)

**Tumor number,** n (%)	

Single	31 (77.5 %)
Multiple	09 (22.5 %)

**Vascular invasion,** n (%)	
Present	08 (20 %)
Absent	32 (80 %)

**Tumor histology (differentiation),** n (%)	

Well-to-moderate	32 (80 %)
Poor	08 (20 %)

**Type of hepatectomy,** n (%)	

Major hepatectomy	14 (35 %)
Minor hepatectomy	26 (65 %)

**Prior treatment,** n (%)	

RFA/TACE	19 (47.5 %)
None	21 (52. 5 %)

**Total bilirubin before resection,** n (%)	

< 1 mg/dl	28 (70 %)
≥ 1 mg/dl	12 (30 %)

**INR before resection,** n (%)	

> 1	30 (75 %)
< 1	10 (25 %)

**AFP before resection,** n (%)	

< 20 ng/ml	25 (62.5 %)
≥ 20 ng/ml	15 (37.5 %)

**Clavien-Dindo postoperative (severe) morbidity,** n (%)	

Absent	35 (87.5 %)
Present	05 (12.5 %)

Min., minimum; max., maximum; n/N, numbers; HBV, hepatitis B virus; HCV, hepatitis C virus; INR, international normalized ratio, AFP, alpha-fetoprotein; PIVKA II, protein induced by vitamin K absence/antagonist-II; TACE, transarterial chemoembolization; RFA, radiofrequency ablation; INR, international normalized ratio.

**Table 2:** Univariable and multivariable analyses with Cox proportional hazard model.

	**Univariable**				**Multivariable**			
**Variables**	**B**	**Exp(B)**	**95**%** CI**	***P* value**	**B**	**Exp(B)**	**95**%** CI**	***P* value**
Age at resection	0.006	1.00	0.94-1.06	0.83				

Sex^#^	-0.03	0.97	0.30-3.03	0.95				

Etiology^#^ (viral/nonviral)	0.34	1.40	0.55-3.58	0.47				

Fibrosis grade^#^ (0-2/3-4)	-0.68	0.50	0.17-1.41	0.19				

Tumor size, cm	-0.82	0.92	0.75-1.12	0.42				

Tumor stage^#^ (I-II/III-IV)	-0.38	0.96	0.30-3.02	0.94				

Tumor Multiplicity^#^	0.48	1.61	0.51-5.09	0.41				

Microvascular invasion^#^	-0.50	0.60	0.13-2.69	0.51				

Histological differentiation^#^ (well-to-moderate/poor)	0.48	1.63	0.36-7.22	0.52				

Prior TACE/RFA^#^	1.36	3.91	1.24-12.32	0.02	1.16	3.18	0.99-10.22	0.05

Type of hepatectomy^#^ (major/minor)	-0.38	0.682	0.21-2.14	0.51				

INR ≥ 1.0^#^	1.77	5.90	0.77-44.94	0.09				

AFP > 20 ng/mL^#^	0.60	1.83	0.66-5.06	0.24				

PIVKA II > 40, mAU/L^#^	-0.25	0.77	0.28-2.14	0.62				

AST, U/L	0.20	1.01	0.99-1.04	0.09				

ALT, U/L	-0.005	0.99	0.97-1.01	0.61				

Albumin, g/L	0.20	1.23	0.46-3.24	0.67				

TB, mg/dL	0.14	1.15	0.34-3.92	0.81				

Preop. platelet count × 10^3^/*μ*L	-0.08	0.92	0.83-1.01	0.10				

Postop. platelet count × 10^3^/*μ*L	-0.15	0.85	0.74-0.99	0.04				

Preop. sPDGF-BB^#^, pg/mL	1.33	3.78	1.20-11.94	0.02	0.84	2.32	0.71-7.54	0.16

Postop. sPDGF-BB^#^, pg/mL	1.87	6.52	1.82-23.24	0.004	1.73	5.64	1.56-20.30	0.008

Clavien-Dindo severe morbidity^#^	-0.77	0.46	0.06-35.0	0.45				

ALT, alanine aminotransferase; AST, aspartate aminotransferase; CI, confidence interval; TB, total bilirubin; AFP, alpha-fetoprotein; PIVKA II, protein induced by vitamin K absence/antagonist-II; TACE, transarterial chemoembolization; RFA, radiofrequency ablation; INR, international normalized ratio; Preop., preoperative; Postop., postoperative; sPDGF-BB, serum platelet derived growth factor-BB; ^#^, categorical variable.

## Data Availability

The data used to support the findings of this study are available from the corresponding author upon request.

## Supplementary Materials

Supplementary Materials Supporting Figure 1: intraplatelet (IP) PDGF-BB concentrations in recurrent and nonrecurrence cases (A) before (PRE OP) and (B) 4 weeks after liver resection (POST OP). IP concentrations are expressed per 10^6^ platelets. Supporting Figure 2: intraplatelet (IP) P-selectin concentrations in recurrent and nonrecurrence cases (A) before (PRE OP) and (B) 4 weeks after liver resection (POST OP). IP concentrations are expressed per 10^6^ platelets.
