# Supplementary material for: Predictive Value of Diminished Serum PDGF-BB after Curative Resection of Hepatocellular Cancer
Source: J Oncol. 2019 Jan 6;2019:1925315. doi: 10.1155/2019/1925315 (PMC6339767; doi:10.1155/2019/1925315)
Supplement: Supplementary Materials — Supporting Figure 1: intraplatelet (IP) PDGF-BB concentrations in recurrent and nonrecurrence cases (A) before (PRE OP) and (B) 4 weeks after liver resection (POST OP). IP concentrations are expressed per 106 platelets. Supporting Figure 2: intraplatelet (IP) P-selectin concentrations in recurrent and nonrecurrence cases (A) before (PRE OP) and (B) 4 weeks after liver resection (POST OP). IP concentrations are expressed per 106 platelets. [file 1925315.f1.pptx]

## Slide 1
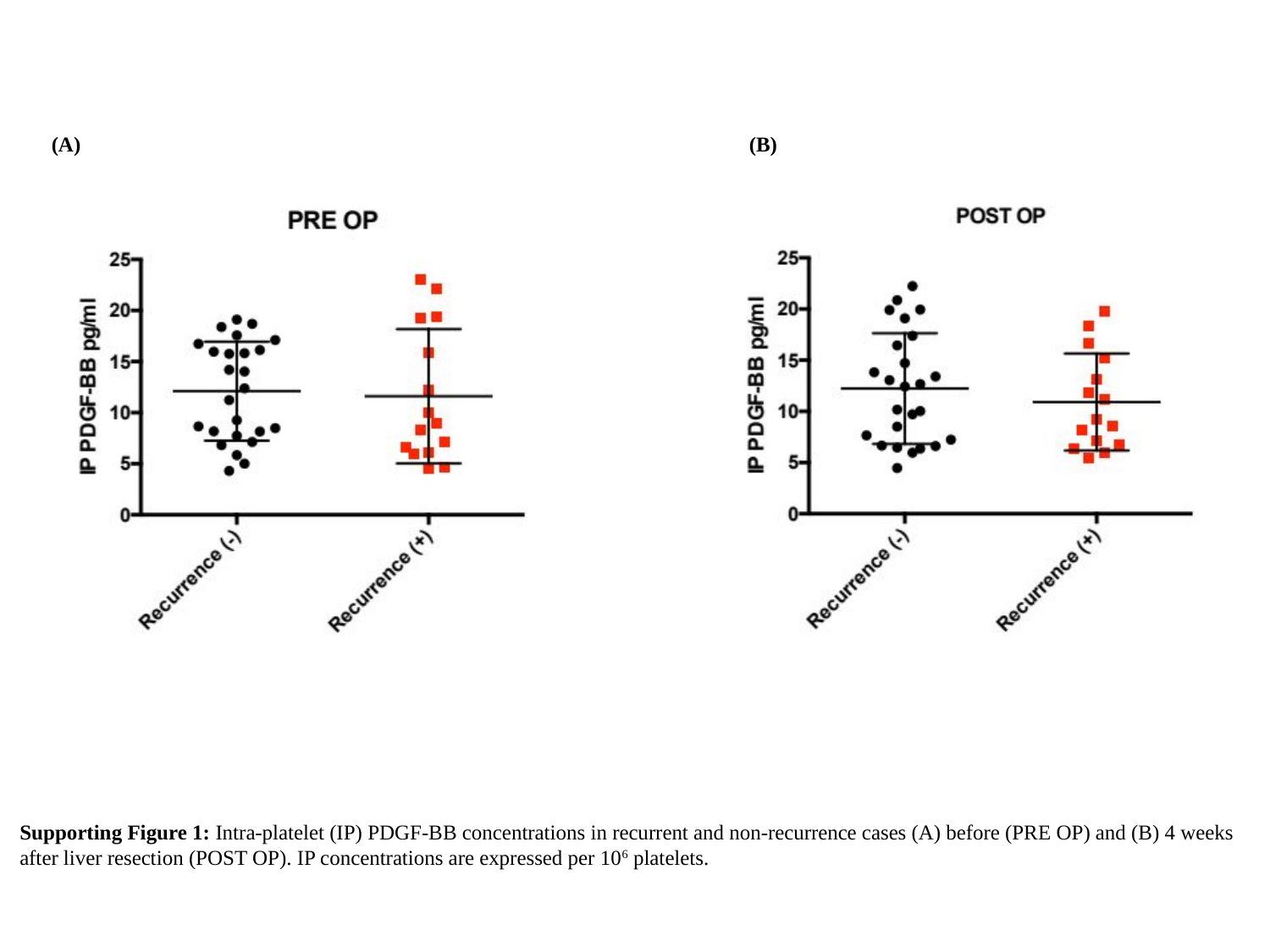

(A)
(B)
Supporting Figure 1: Intra-platelet (IP) PDGF-BB concentrations in recurrent and non-recurrence cases (A) before (PRE OP) and (B) 4 weeks
after liver resection (POST OP). IP concentrations are expressed per 106 platelets.

## Slide 2
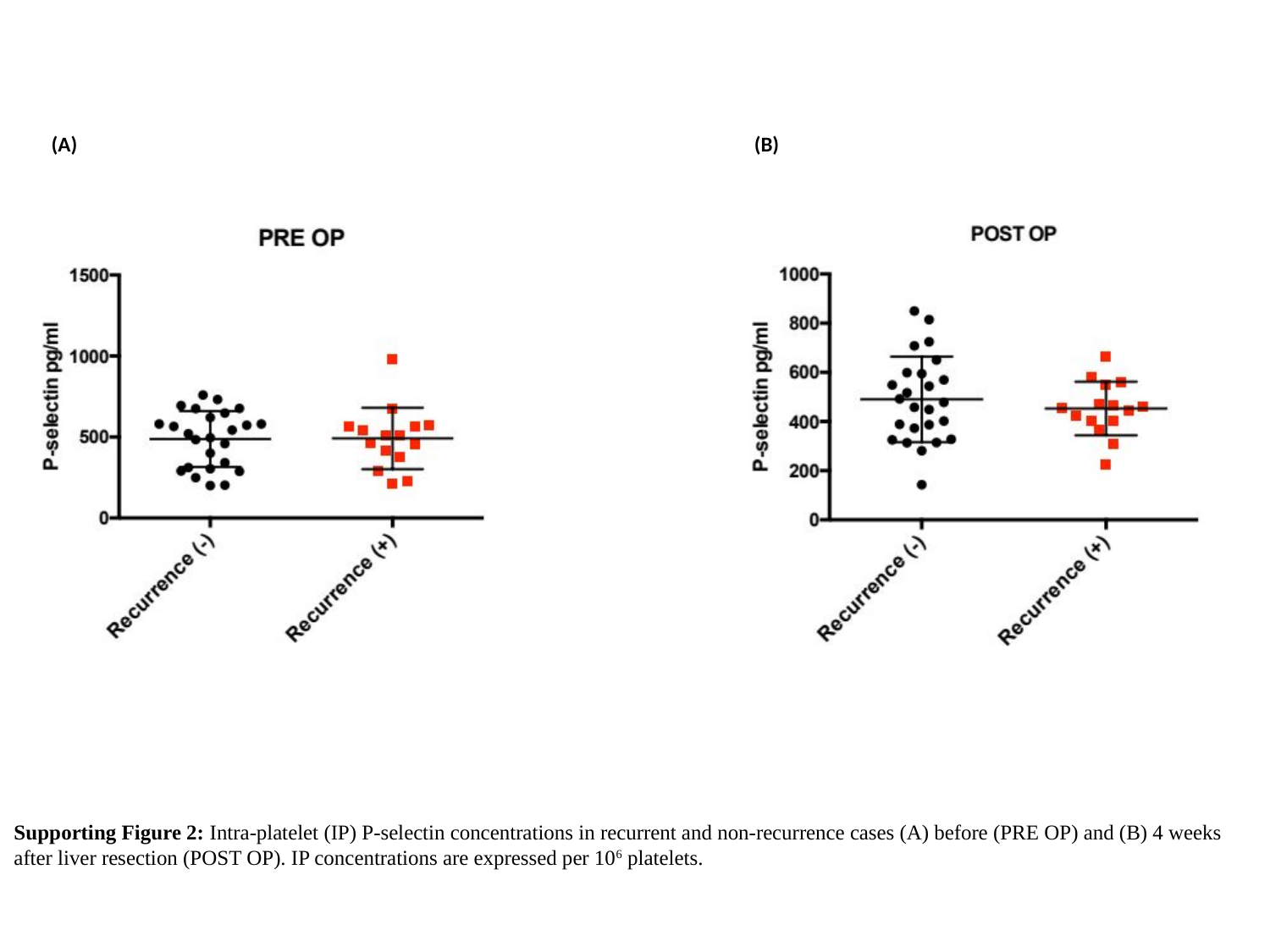

(A)
(B)
Supporting Figure 2: Intra-platelet (IP) P-selectin concentrations in recurrent and non-recurrence cases (A) before (PRE OP) and (B) 4 weeks
after liver resection (POST OP). IP concentrations are expressed per 106 platelets.
